# Supplementary material for: Correlation between Dietary Intake of Vitamins and Oral Health Behaviors: A Cross-Sectional Study
Source: Int J Environ Res Public Health. 2023 Mar 23;20(7):5243. doi: 10.3390/ijerph20075243 (PMC10094008; doi:10.3390/ijerph20075243)
Supplement: Supplementary file 1 [file ijerph-20-05243-s001.zip › ijerph-2265177-supplementary.pdf]

Supplementary Materials

Table S1. Correlation among the vitamins intakes.

	Vitamins	1	2	3	4	5	6	7
1	Vitamin A	1.000						
2	Vitamin B_2	0.466 **	1.000					
3	Vitamin B_6	0.520 **	0.546 **	1.000				
4	Niacin	0.337 **	0.417 **	0.730 **	1.000			
5	Folic acid	0.615 **	0.594 **	0.793 **	0.557 **	1.000		
6	Vitamin E	0.522 **	0.509 **	0.553 **	0.564 **	0.548 **	1.000	
7	Vitamin K	0.482 **	0.431 **	0.590 **	0.426 **	0.675 **	0.505 **	1.000

**: $p < 0.01$.

Table S2. Correlation between %BOP and intake of vitamins.

Vitamins	Whole (n = 218)	Nonsmoking (n = 174)	Smoking (n = 44)
Vitamin A	0.00	0.02	−0.02
Vitamin B_1	−0.09	−0.12	0.04
Vitamin B_2	−0.07	−0.10	0.07
Vitamin B_6	−0.02	−0.01	−0.01
Vitamin B_{12}	−0.09	−0.09	−0.09
Folic Acid	0.06	0.09	0.00
Niacin	−0.08	−0.07	−0.11
Vitamin C	−0.01	0.00	0.05
Vitamin D	0.05	0.07	0.01
Vitamin E	−0.06	−0.04	−0.14
Vitamin K	0.07	0.06	0.13

Table S3. Differences of food intakes between two groups divided based on oral health behavior with interdental cleaning or tongue brushing.

Food (*m*, g/day)	Oral Health Behavior with Interdental Cleaning or Tongue Brushing		*p*-Value
	Yes (*n* = 133)	No (*n* = 85)	
Beans including processed food	31.7 (0–370)	32.1 (0–375)	0.388
Green and yellow vegetable	86.8 (0–496)	65.6 (0–422)	0.009
Milk, dairy products	140.0 (0–626)	60.0 (0–628)	0.069
Grains	375.0 (39–857)	400.0 (155–1180)	0.136
